# The Role of CODA and GPR Programs on Comprehensive Oral Health Care for Persons With Disabilities

**DOI:** 10.1111/scd.70147

**Published:** 2026-02-07

**Authors:** Miriam R. Robbins

**Affiliations:** ^1^ Clinical Oral Medicine University of Pennsylvania School of Dental Medicine Philadelphia Pennsylvania USA; ^2^ Clinical Preventive and Restorative Dentistry University of Pennsylvania School of Dental Medicine Philadelphia Pennsylvania USA

**Keywords:** dental accreditation, disabilities, general practice residencies, graduate dental education, special needs

## Abstract

The landscape of dental education and clinical practice has undergone significant evolution in recent decades, driven by increasing recognition of health disparities and the evolving needs of diverse patient populations. Central to this transformation is the role of the Commission on Dental Accreditation (CODA) in establishing rigorous postdoctoral standards for dental education. Concurrently, General Practice Residency (GPR) programs have emerged as pivotal training models that prepare dental practitioners to deliver high‐quality, comprehensive oral health care—especially for persons with disabilities and medically complex conditions. This article examines CODA's historical and current influence on dental education, highlights the critical training components of GPR programs, and discusses how these initiatives contribute to enhanced care for underserved populations, improving access and quality of oral health care for vulnerable populations

## 1 Introduction

Oral health is inextricably linked to overall well‐being, yet disparities persist in access to quality dental care, especially among individuals with disabilities and complex medical conditions. Despite significant advancements in clinical techniques and technology, many patients encounter physical, financial, and systemic barriers that limit their access to needed care. In recent years, there has been growing recognition of the importance of equitable health care for all populations. There has been a growing emphasis on establishing rigorous educational standards for dental practitioners—standards that not only foster clinical excellence but also promote compassionate, patient‐centered care. Central to this transformation is the Commission on Dental Accreditation (CODA), whose mission is to serve the public by ensuring that dental education programs meet and exceed national quality standards. CODA's influence has extended into every facet of dental education, including the critical postdoctoral phase. It is within this realm that General Practice Residency (GPR) programs have emerged as vital training environments, providing advanced clinical experience and interdisciplinary collaboration that are essential for training practitioners who are both clinically proficient and empathetic in managing diverse patient needs. This training model is particularly crucial for preparing dental practitioners to care for patients with disabilities, who often require modified treatment protocols, effective communication strategies, and a comprehensive understanding of their multifaceted health needs.

Ultimately, the integration of CODA standards with the comprehensive training provided by GPR programs forms the backbone of a modern, inclusive dental education system that has the potential to reduce health disparities and improve the quality of life for some of society's most vulnerable members. Despite these educational advances, people with disabilities still confront substantial physical, financial, and systemic obstacles to receiving dental care. Provider shortages, inaccessible facilities, and limited insurance coverage continue to suppress utilization and worsen outcomes. Robust CODA standards and expertly structured GPR programs are therefore indispensable catalysts; by cultivating practitioners who can navigate hospital systems, leverage integrated EHRs, and tailor care plans, they directly mitigate some of these barriers and measurably improve patient satisfaction and oral‑health status [1, 2].

## 2 Historical Evolution of CODA and Its Role in Dental Education

### 2.1 Establishment and Early Mandate of CODA

CODA was established in 1975 under the auspices of the American Dental Association (ADA) as a response to growing public concerns about the quality and consistency of dental education. In its earliest years, CODA's primary objective was to ensure that dental schools in the United States produced graduates who possessed the necessary technical and clinical competencies to practice dentistry safely. During this formative period, the focus was on measurable clinical skills and the ability to perform routine procedures.

As dental education evolved, however, it became apparent that technical proficiency alone was insufficient for addressing the complex healthcare needs of a diversifying population. Over time, CODA's mandate broadened to encompass additional areas such as ethical practice, cultural competence, patient safety, and the management of patients with special needs (defined as patients whose medical, physical, psychological or social circumstances require modifications to standard dental care) [3]. This shift reflected broader societal changes, including an increased awareness of health disparities and the recognition that underserved populations—particularly individuals with disabilities—often faced significant challenges in accessing quality dental care.

### 2.2 Shifting Focus toward Inclusivity and Special Needs

During the late 1990s and early 2000s, CODA began to explicitly incorporate training requirements for the management of patients with special needs into its accreditation criteria. Prior to this period, dental education had largely been focused on technical and procedural competencies. However, evidence began to accumulate showing that many patients, especially those with disabilities, were not receiving appropriate care due to a lack of provider training in managing complex medical and behavioral issues. CODA responded by revising its accreditation standards to include specific provisions that mandated exposure to and training in the treatment of patients with special health care needs. Since its inception, Standard 2–25 in predoctoral education has evolved to now requiring that dental curricula include adequate clinical exposure in the assessment and management of patients with disabilities to ensure that graduates are not only clinically adept but also prepared to provide empathetic, patient‑centered care. This historical evolution of CODA's standards laid the groundwork for subsequent changes in postdoctoral education, where the emphasis on managing medically complex patients became even more pronounced [4].

### 2.3 CODA's Ongoing Commitment to Continuous Improvement

In recent years, CODA has maintained its commitment to continuously improving the quality of dental education by regularly updating its accreditation standards. This iterative process involves rigorous self‐study reports, on‐site evaluations, and continuous monitoring to ensure compliance with established benchmarks. The dynamic nature of CODA's standards reflects an ongoing recognition that the healthcare environment is continually evolving. New technologies, changing patient demographics, and emerging research all necessitate periodic revisions of the standards to ensure that dental education remains current and effective.

The transformation of CODA's role from a body focused solely on technical competencies to one that also emphasizes cultural competence, ethical practice, and interdisciplinary collaboration is significant. These changes have had a profound impact on the curricula of both predoctoral and postdoctoral programs, fostering an educational environment that is more holistic and responsive to the needs of diverse patient populations.

## 3 CODA Standards in Postdoctoral Dental Education

### 3.1 Defining the Scope of Postdoctoral Training

Postdoctoral dental education, which includes programs such as GPR programs and Advanced Education in General Dentistry (AEGD) programs, serves as the bridge between dental school and independent practice. The purpose of postdoctoral training is to provide dental graduates with advanced clinical skills, deepen their understanding of complex patient care, and enhance their ability to manage challenging cases. CODA plays a critical role in defining the educational outcomes expected from these programs by setting rigorous standards that emphasize both clinical proficiency and broader professional competencies.

### 3.2 Emphasis on Clinical Proficiency and Advanced Treatment Modalities

An integral part of the standards for postdoctoral dental education is the requirement for extensive clinical training. This involves not only routine dental procedures but also the management of complex cases involving medically compromised patients. Residents in GPR programs are expected to master advanced treatment modalities, including the use of sedation, pain management techniques, and surgical interventions, all within the context of treating patients with intricate medical histories. The focus on clinical proficiency ensures that graduates are well‐prepared to adapt their treatment protocols to the unique needs of patients with disabilities.

The integration of simulation‐based training and hands‐on clinical experiences in hospital environments has become a cornerstone of postdoctoral programs. These immersive training experiences allow residents to gain practical skills in real‐world settings, where they encounter a diverse patient population. Research has shown that dental programs with robust clinical training components produce graduates who are more adept at managing complex cases, ultimately leading to improved patient outcomes and higher levels of patient satisfaction [5].

### 3.3 Fostering Interdisciplinary Collaboration and Cultural Competence

In today's interconnected healthcare landscape, the delivery of quality dental care often necessitates close collaboration among various healthcare professionals. CODA's standards emphasize the importance of interdisciplinary training, mandating that postdoctoral programs incorporate rotations in medical specialties such as internal medicine, pediatrics, and geriatrics. These interdisciplinary experiences enable residents to gain a comprehensive understanding of how systemic health conditions can influence oral health, and they foster an environment of teamwork and collaboration that is essential for holistic patient care [6].

Alongside clinical and interdisciplinary training, CODA also stresses the importance of cultural competence and effective communication. With the increasing diversity of patient populations, dental practitioners must be prepared to navigate cultural and linguistic differences while providing empathetic care. CODA requires that educational programs integrate modules on communication, cultural sensitivity, and ethical practice into their curricula. This approach not only prepares residents to manage the clinical aspects of care but also equips them to address the social determinants of health that can impact patient outcomes.

### 3.4 The Accreditation Process as a Driver for Innovation

CODA's accreditation process serves as a powerful incentive for continuous innovation in dental education. Programs that fail to meet established standards risk losing accreditation, which motivates institutions to adopt new teaching methodologies and invest in advanced technologies. Many dental schools have responded by integrating virtual simulation platforms, telehealth modules, and interdisciplinary case conferences into their curricula. These innovations are designed to enhance learning experiences, increase clinical proficiency, and ultimately lead to better patient care.

Recent studies have provided evidence that adherence to updated CODA standards is correlated with improved clinical outcomes and higher levels of patient satisfaction. Dental programs with rigorous accreditation practices produced graduates who were better prepared to manage the needs of medically complex patients. [7] Interdisciplinary training has been shown to have a positive impact on comprehensive treatment planning and patient outcomes [8]. These findings underscore the transformative effect of CODA's standards on postdoctoral dental education.

### 3.5 Overview of GPR Programs

GPR programs represent one of the primary pathways through which dental graduates receive advanced clinical training. Typically lasting one to two years, GPR programs are designed to provide an immersive, hospital‐based experience that bridges the gap between dental school and independent practice. Unlike traditional dental settings where treatment may be limited to routine procedures, GPR programs expose residents to a wide spectrum of clinical scenarios—including emergency care, surgical interventions, and the management of patients with chronic or complex medical conditions.

GPR programs are uniquely positioned to address the growing demand for dentists who are not only technically proficient but also skilled in managing the multifaceted needs of patients with disabilities and medical complexities. The advanced clinical training provided in GPR settings emphasizes comprehensive patient care, incorporating both the technical aspects of dentistry and the nuanced considerations required for treating patients with disabilities [9].

### 3.6 Clinical Training in Hospital Environments

A defining characteristic of GPR programs is the extensive clinical exposure provided in hospital settings. In these environments, residents work alongside multidisciplinary teams that include physicians, nurses, and other healthcare professionals. Hospital dentistry offers a dynamic setting where residents are confronted with complex cases that require a nuanced understanding of both dental and medical considerations. The hospital setting provides access to advanced diagnostic tools, sedation services, and comprehensive patient monitoring systems that are not typically available in traditional dental offices. Equally important, residents gain daily experience navigating an integrated electronic health record (EHR) system, which prepares them to retrieve pertinent medical information, document dental interventions, and communicate seamlessly with the broader care team [10].

During their rotations in hospitals, residents are exposed to patients with a broad range of medical conditions, such as cardiovascular disease, diabetes, neurological disorders, and various chronic illnesses. This exposure is critical for teaching residents how to modify standard dental procedures to ensure the safety and comfort of medically compromised patients. For example, residents learn to tailor treatment protocols for patients on anticoagulant therapy, manage the dental needs of diabetic patients who are at increased risk for periodontal disease, and address the unique challenges posed by patients with cognitive impairments. The hands‐on clinical experience in hospital environments instills a level of competence and confidence that is essential for managing complex cases.

The incorporation of telehealth technologies during hospital rotations further enhances the training experience. By participating in remote consultations and virtual patient monitoring, residents learn how to extend the continuum of care beyond the confines of the hospital setting. This integration of technology is particularly beneficial for patients with mobility challenges or those residing in rural areas, as it facilitates timely interventions and ensures continuity of care. Studies have demonstrated that the use of telehealth in dental training not only improves clinical outcomes but also enhances patient satisfaction by making care more accessible and responsive [11].

### 3.7 Interdisciplinary Rotations and Collaborative Learning

Beyond hospital‐based training, GPR programs emphasize the importance of interdisciplinary rotations. Residents typically rotate through various medical departments, including pediatrics, internal medicine, and geriatrics, to gain a comprehensive understanding of how systemic health conditions affect oral health. These interdisciplinary experiences are instrumental in teaching residents how to integrate dental and medical care. For instance, a rotation in pediatrics exposes residents to the challenges of treating children with developmental disabilities, while a rotation in internal medicine provides insight into the management of chronic conditions such as hypertension and diabetes.

Collaborative learning is a core component of these interdisciplinary rotations. Residents participate in joint case conferences, clinical rounds, and multidisciplinary team meetings where they discuss complex patient cases with professionals from various specialties. These collaborative sessions foster an environment of shared learning, where residents are encouraged to consider diverse perspectives and develop integrated treatment plans. The benefits of such an approach are twofold: residents not only refine their clinical decision‐making skills but also develop the interpersonal and communication competencies necessary for effective teamwork. Interdisciplinary training significantly enhances residents’ abilities to diagnose and manage complex cases, leading to improved clinical outcomes and greater patient satisfaction [12, 13].

### 3.8 Development of Communication and Interpersonal Skills

Effective communication is paramount in the delivery of high‐quality dental care, particularly when treating patients with disabilities. GPR programs place substantial emphasis on cultivating the communication and interpersonal skills of their residents. Training methodologies include role‐playing exercises, simulated patient encounters, and interdisciplinary team meetings—all designed to enhance the residents’ ability to interact with patients in a compassionate and empathetic manner.

Residents are taught to tailor their communication strategies to meet the unique needs of each patient. For example, when treating patients with cognitive impairments or sensory limitations, residents learn to use simplified language, visual aids, and nonverbal cues to ensure that patients fully understand the treatment process. This patient‐centered approach to communication not only builds trust and rapport but also facilitates better treatment adherence and reduces patient anxiety. It. underscores the importance of effective communication in improving patient outcomes, demonstrating that enhanced communication skills are directly associated with higher levels of patient satisfaction and overall treatment success [14].

### 3.9 Advanced Training in Treatment Planning for Complex Cases

The formulation of comprehensive treatment plans for medically complex patients represents one of the most challenging aspects of dental practice. GPR programs dedicate significant portions of their curricula to developing residents’ competencies in treatment planning. Through participation in case conferences, simulation exercises, and one‐on‐one mentoring sessions, residents learn to integrate multiple sources of information (including detailed medical histories, social determinants of health, and patient preferences) into a coherent and individualized treatment plan.

This aspect of training emphasizes critical thinking and problem‐solving skills, enabling residents to anticipate potential complications and develop contingency plans. Mentorship from experienced faculty plays a crucial role in refining these skills, as residents are provided with constructive feedback and guidance on optimizing their treatment strategies. Studies have shown that a structured, hands‐on approach to treatment planning significantly improves the quality of care delivered to patients with disabilities, as it fosters a deeper understanding of the complex interplay between various health factors [15].

## 4 Training and Competency in Patients With Special Needs

### 4.1 The Imperative for Specialized Training

The provision of oral health care for individuals with disabilities necessitates specialized training that goes beyond the scope of routine dental education. Patients with special needs often present with unique challenges, such as behavioral issues, heightened anxiety, and complex medical histories that require careful management. Inadequate training in these areas can result in misdiagnosis, inappropriate treatment protocols, and ultimately, poorer health outcomes. To address these challenges, GPR programs and other postdoctoral training initiatives have increasingly focused on developing both clinical and interpersonal competencies specific to the delivery of care for these patients.

### 4.2 Hands‐On Clinical Experience in Hospital Settings

One of the most effective ways to prepare dental practitioners for the complexities of treatment of patients with disabilities and medical complexities is through immersive, hands‐on clinical experience. Hospital dentistry provides a setting in which residents are exposed to a wide variety of cases that encompass the full spectrum of medical complexity. In these settings, residents have the opportunity to work with patients who have significant cognitive, physical, or sensory limitations, learning first‐hand how to adapt standard dental procedures to accommodate their needs.

This training is particularly beneficial for instilling confidence in residents, as it allows them to manage challenging cases under the supervision of experienced faculty. The practical experience gained in hospital settings is invaluable, as it prepares residents to handle emergencies, modify treatment protocols, and effectively manage complications that may arise due to underlying medical conditions. Research indicates that residents who receive extensive clinical training in hospital environments are better equipped to treat patients with special needs, leading to enhanced clinical outcomes and improved patient satisfaction [16].

### 4.3 Interdisciplinary Education and Its Impact on Competency

Interdisciplinary education is a cornerstone of effective training in hospital dentistry. Recognizing that the oral health of medically complex patients is closely linked to their overall health, GPR programs have incorporated rotations in various medical disciplines into their curricula. These interdisciplinary experiences provide residents with a broader understanding of the ways in which systemic conditions—such as diabetes, cardiovascular disease, and neurological disorders—affect dental treatment.

In these rotations, residents work alongside professionals from multiple fields, including medicine, nursing, and mental health. The exchange of knowledge that occurs in these collaborative environments enhances residents’ ability to develop integrated treatment plans that address both dental and systemic health concerns. Interdisciplinary collaboration fosters a comprehensive approach to patient care that is critical for managing the complex needs of special care patients [17].

### 4.4 Development of Behavioral Management and Communication Strategies

An essential component of training is the development of behavioral management and communication strategies. Many patients with disabilities experience significant anxiety and may have difficulty communicating their needs effectively. In response, GPR programs emphasize the importance of patient‐centered communication, equipping residents with techniques designed to reduce anxiety and facilitate clear, empathetic interactions.

Residents are trained in various methods for managing patient behavior, including desensitization techniques, the use of positive reinforcement, and strategies for involving caregivers in the treatment process. These skills are not only crucial for improving patient cooperation during dental procedures but also for ensuring that patients leave the dental chair with a positive impression of their care experience. Enhanced communication skills have been shown to correlate with higher levels of patient satisfaction and better overall treatment outcomes [18].

### 4.5 Ethical Considerations and Professional Responsibilities

In addition to clinical and communication competencies, training must also address the ethical and professional responsibilities of dental practitioners. Residents are taught to recognize the unique challenges faced by patients with disabilities and to approach their care with sensitivity, respect, and a commitment to equity. The ethical framework provided in GPR programs emphasizes the importance of treating all patients with dignity and advocating for those who may be vulnerable or marginalized.

This aspect of training is critical, as it not only influences the quality of care provided but also shapes the professional identity of future dental practitioners. By instilling a strong ethical foundation early in their training, GPR programs help ensure that graduates are not only clinically proficient but also deeply committed to the principles of social justice and health equity. Such a commitment is essential for addressing the systemic disparities that continue to impact access to dental care for special needs populations.

### 4.6 Integration of Telehealth and Technological Innovations

Technological advancements have begun to play an increasingly important role in special care dentistry. The integration of telehealth into dental education represents a significant innovation that has the potential to expand access to care for patients with disabilities. Telehealth enables residents to conduct remote consultations, perform virtual follow‐ups, and even carry out certain aspects of diagnostic assessments. This approach is especially beneficial for patients with mobility challenges or those residing in remote areas, as it facilitates continuity of care without the need for in‐person visits. Mastery of integrated EHR platforms further enhances residents’ ability to acquire and apply patient information in real time, ensuring seamless coordination of care [19].

GPR programs that incorporate telehealth training provide residents with the technical skills required to use digital platforms effectively, as well as the communication strategies necessary for remote patient engagement. The adoption of telehealth not only improves access to care but also represents a forward‐thinking approach to the evolving landscape of healthcare delivery. Studies have highlighted the positive impact of telehealth on patient satisfaction and clinical outcomes, further underscoring its importance in modern dental practice [20].

### 4.7 The Role of Well‐Trained Practitioners in Overcoming Barriers

Well‐trained dental practitioners are essential for addressing the multifaceted barriers that impede access to quality care for patients with disabilities. Training that focuses on cultural competence, advanced management techniques, and effective communication can empower practitioners to create a more welcoming and supportive environment. When dental professionals are confident in their abilities to modify treatment protocols, adapt to the unique needs of special care patients, and collaborate with other healthcare providers, they are better positioned to overcome systemic challenges and improve access to care [21].

In addition to clinical competence, commitment to advocacy is critical. Dental practitioners who have been thoroughly trained in providing care to patients with disabilities are often more willing to advocate for systemic changes that benefit vulnerable populations. This may include working with policymakers to expand dental coverage under public insurance programs, improving accessibility standards in dental offices, or developing community‐based outreach programs to extend care to underserved areas. The positive correlation between robust training and improved access to care has been well documented in the literature, suggesting that investments in comprehensive education ultimately yield significant public health benefits [22].

### 4.8 The Impact of Training on Patient Outcomes and Satisfaction

The comfort and confidence of dental practitioners have a direct influence on patient outcomes. When practitioners are well‐trained and knowledgeable about the needs of patients with disabilities, they are more likely to deliver high‐quality care, establish strong patient–practitioner relationships, and reduce patient anxiety during treatment [23, 24]. Research has demonstrated that patients who feel understood and supported by their dental providers are more likely to adhere to treatment plans, attend follow‐up appointments, and experience overall better oral health outcomes [25, 26].

Furthermore, effective communication and personalized care are critical for ensuring that patients with special needs are treated with dignity and respect. The integration of comprehensive training in GPR programs—encompassing both clinical and interpersonal skills—contributes to improved patient satisfaction and higher levels of trust [27]. This, in turn, enhances treatment adherence and leads to more favorable clinical results [28].

## 5 Future Directions and Recommendations

### 5.1 Enhancements in GPR Program Curricula

As the needs of patients with disabilities continue to evolve, so too must the educational strategies employed by GPR programs. Future enhancements should include an even greater emphasis on emerging technologies such as telehealth, which can help bridge geographic and physical barriers to care. [29] Training modules focused on virtual consultations, remote monitoring, and digital diagnostics are likely to become increasingly important in ensuring that dental care remains accessible to all. Emphasizing EHR proficiency positions residents as effective contributors within hospital‐based collaborative entities [30].

Furthermore, there is a need for continuous improvement in the interdisciplinary aspects of GPR training. Formal partnerships between dental programs and other medical disciplines should be expanded, with more opportunities for cross‐training and collaborative case management. Programs should also consider incorporating advanced simulation tools that mimic real‐life scenarios involving patients with complex medical histories and disabilities. These innovations will not only improve clinical competence but also foster a culture of continuous learning and adaptation [31, 32]

### 5.2 Policy Advocacy and Systemic Change

To fully realize the promise of equitable oral health care for persons with disabilities, advocacy efforts must extend beyond the confines of dental education. Stakeholders—including dental educators, professional associations, and policymakers—must work together to address systemic barriers that hinder access to care. This includes advocating for improved reimbursement rates for medically necessary dentistry, expanding insurance coverage for comprehensive dental services, and enforcing accessibility standards in all dental facilities.

Recent policy initiatives in some states have begun to address these issues, but further legislative and regulatory efforts are needed at the national level. By leveraging the collective expertise of the dental community and the data generated from well‐run GPR programs, stakeholders can build a compelling case for systemic reform that benefits both patients and practitioners [33]

### 5.3 Research and Innovation Priorities

Future research should focus on assessing the long‐term impact of CODA‐accredited GPR programs on patient outcomes, particularly among populations with disabilities. Longitudinal studies tracking graduates from these programs can provide valuable insights into the efficacy of current training methods and highlight areas for improvement [34]. Additionally, research into the integration of telehealth and other digital tools in dental practice could pave the way for more innovative and accessible care models.

Opportunities for innovation in care delivery should also be a priority. For example, pilot projects that combine traditional clinical care with community‐based outreach programs may offer new models for extending dental services to underserved populations. Research into these innovative models can inform best practices and lead to the development of scalable solutions that can be implemented across diverse healthcare settings.

### 5.4 Strengthening the Evidence Base

Building a robust evidence base is essential for driving continuous improvement in dental education and practice. Future studies should explore the direct correlations between enhanced postdoctoral training—under the auspices of CODA standards—and measurable improvements in oral health outcomes for persons with disabilities and medical complexities. Such research will not only validate the current training paradigms but also help justify further investments in specialized dental education programs.

In addition, qualitative research that captures the experiences of patients, families, and dental practitioners can offer deeper insights into the factors that influence care quality. Understanding the nuances of patient–practitioner interactions and the challenges faced by individuals with disabilities can inform the development of more tailored training programs and practice guidelines.

### 5.5 Embracing Global Perspectives

While this article focuses on the U.S. dental education system, it is important to recognize that the challenges of providing equitable dental care for persons with disabilities are global in scope. International collaboration and knowledge exchange can play a crucial role in identifying innovative solutions and best practices. Dental educators and researchers should seek opportunities to engage with global partners, participate in international conferences, and contribute to comparative studies that examine how different healthcare systems address similar challenges. Such collaborations can enrich the training experience and promote a more holistic approach to dental care worldwide.

## 6 Conclusion

The evolution of dental education over the past several decades has been profoundly influenced by the work of CODA and the development of GPR programs. By establishing rigorous postdoctoral standards, CODA has ensured that dental education programs are held to the highest levels of quality, preparing graduates to meet the complex challenges of modern dental practice. These standards emphasize clinical proficiency, interdisciplinary collaboration, cultural competence, and the ethical treatment of all patients—principles that are particularly vital when caring for individuals with disabilities and medically complex conditions.

GPR programs, with their emphasis on hands‐on clinical experience in hospital settings, interdisciplinary rotations, and comprehensive treatment planning, provide an ideal environment for fostering the skills and confidence needed to deliver high‐quality, patient‐centered care. [35] For patients with disabilities, who often face a multitude of barriers—from physical accessibility issues to financial constraints—such specialized training is essential. Well‐trained practitioners are better equipped to adapt treatment protocols, communicate effectively, and advocate for systemic changes that improve access to care.

Looking ahead, the continued evolution of CODA standards and GPR programs will be critical in addressing the ever‐changing landscape of healthcare. Embracing technological innovations such as telehealth, strengthening interdisciplinary partnerships, and advocating for systemic policy changes are all necessary steps toward ensuring that dental care remains inclusive, equitable, and effective. Ongoing research and global collaboration will further reinforce the importance of specialized dental training and help drive continuous improvements in both education and clinical practice. Consistent evidence links robust EHR‐enabled care coordination with improved satisfaction and measurable gains in oral‑health outcomes [36]

In summary, the commitment to excellence embodied by CODA‐accredited GPR programs has a transformative impact on the dental profession. By equipping practitioners with the necessary technical, communicative, and ethical skills, these programs not only enhance the overall quality of dental care but also play a critical role in reducing health disparities among vulnerable populations. The future of dental care for persons with disabilities and medically complexities depends on the continued dedication of educators, practitioners, and policymakers to advance training standards, foster innovation, and build a more inclusive healthcare system.

## Conflicts of Interest

The author declares no conflicts of interest.
